# Supplementary material for: Modeling glioblastoma heterogeneity as a dynamic network of cell states
Source: Mol Syst Biol. 2021 Sep 16;17(9):e10105. doi: 10.15252/msb.202010105 (PMC8444284; doi:10.15252/msb.202010105)
Supplement: Supplementary file 6 — Source Data for Figure 5 [file MSB-17-e10105-s004.zip › Figure5A_sourcedata/GSEA_3017/nosigdb_stateD.GseaPreranked.1622795010397/FELDMAN_2020_S-G2.html]

Details for gene set FELDMAN\_2020\_S-G2[GSEA]

|  || Dataset | state23017 |
| Phenotype | NoPhenotypeAvailable |
| Upregulated in class | na\_neg |
| GeneSet | FELDMAN\_2020\_S-G2 |
| Enrichment Score (ES) | -0.55628276 |
| Normalized Enrichment Score (NES) | -3.1879551 |
| Nominal p-value | 0.0 |
| FDR q-value | 0.0 |
| FWER p-Value | 0.0 |
Table: GSEA Results Summary

  

Fig 1: Enrichment plot: FELDMAN\_2020\_S-G2      
 Profile of the Running ES Score & Positions of GeneSet Members on the Rank Ordered List

  

| PROBE | GENE SYMBOL | GENE\_TITLE | RANK IN GENE LIST | RANK METRIC SCORE | RUNNING ES | CORE ENRICHMENT || 1 | PTTG1 |  |  | 14 | 0.490 | -0.0068 | No |
| 2 | RAB13 |  |  | 43 | 0.367 | -0.0270 | No |
| 3 | SMS |  |  | 45 | 0.358 | -0.0240 | No |
| 4 | BTG1 |  |  | 50 | 0.350 | -0.0237 | No |
| 5 | PHLDA1 |  |  | 59 | 0.336 | -0.0269 | No |
| 6 | SOX4 |  |  | 91 | 0.298 | -0.0505 | No |
| 7 | IER2 |  |  | 101 | 0.290 | -0.0552 | No |
| 8 | TMEM158 |  |  | 120 | 0.275 | -0.0677 | No |
| 9 | BIRC5 |  |  | 130 | 0.271 | -0.0726 | No |
| 10 | TUBA1A |  |  | 148 | 0.259 | -0.0845 | No |
| 11 | MKI67 |  |  | 206 | -0.254 | -0.1310 | No |
| 12 | PXMP2 |  |  | 233 | -0.257 | -0.1507 | No |
| 13 | PLD3 |  |  | 240 | -0.258 | -0.1531 | No |
| 14 | FTL |  |  | 271 | -0.261 | -0.1762 | No |
| 15 | TRIP13 |  |  | 294 | -0.263 | -0.1924 | No |
| 16 | GPM6B |  |  | 314 | -0.265 | -0.2060 | No |
| 17 | EXOSC8 |  |  | 347 | -0.268 | -0.2308 | No |
| 18 | RNASEH2B |  |  | 370 | -0.270 | -0.2468 | No |
| 19 | CARHSP1 |  |  | 405 | -0.274 | -0.2733 | No |
| 20 | TUBB |  |  | 410 | -0.274 | -0.2738 | No |
| 21 | CHCHD3 |  |  | 420 | -0.276 | -0.2786 | No |
| 22 | SNRNP40 |  |  | 502 | -0.285 | -0.3455 | No |
| 23 | SIVA1 |  |  | 567 | -0.293 | -0.3977 | No |
| 24 | RFC5 |  |  | 568 | -0.293 | -0.3945 | No |
| 25 | RFC4 |  |  | 575 | -0.295 | -0.3965 | No |
| 26 | BTG3 |  |  | 581 | -0.296 | -0.3976 | No |
| 27 | TEX30 |  |  | 592 | -0.298 | -0.4031 | No |
| 28 | RFC2 |  |  | 607 | -0.302 | -0.4119 | No |
| 29 | MCM3 |  |  | 625 | -0.305 | -0.4233 | No |
| 30 | DCK |  |  | 631 | -0.307 | -0.4243 | No |
| 31 | ENC1 |  |  | 643 | -0.309 | -0.4304 | No |
| 32 | PRC1 |  |  | 645 | -0.310 | -0.4279 | No |
| 33 | DNMT1 |  |  | 659 | -0.312 | -0.4358 | No |
| 34 | TMPO |  |  | 688 | -0.317 | -0.4566 | No |
| 35 | RAD23A |  |  | 696 | -0.319 | -0.4592 | No |
| 36 | S100B |  |  | 705 | -0.320 | -0.4626 | No |
| 37 | KIF4A |  |  | 706 | -0.320 | -0.4591 | No |
| 38 | SDCBP |  |  | 734 | -0.327 | -0.4790 | No |
| 39 | WDR34 |  |  | 752 | -0.331 | -0.4901 | No |
| 40 | NDFIP1 |  |  | 805 | -0.346 | -0.5313 | No |
| 41 | BRCA2 |  |  | 808 | -0.347 | -0.5293 | No |
| 42 | H2AFX |  |  | 810 | -0.347 | -0.5264 | No |
| 43 | CENPM |  |  | 818 | -0.349 | -0.5286 | No |
| 44 | MAD2L1 |  |  | 819 | -0.350 | -0.5248 | No |
| 45 | BUB3 |  |  | 826 | -0.351 | -0.5262 | No |
| 46 | SHCBP1 |  |  | 844 | -0.356 | -0.5371 | No |
| 47 | ARL6IP6 |  |  | 854 | -0.358 | -0.5409 | No |
| 48 | TFDP1 |  |  | 860 | -0.359 | -0.5414 | No |
| 49 | CDCA4 |  |  | 870 | -0.363 | -0.5452 | No |
| 50 | PRKDC |  |  | 871 | -0.363 | -0.5413 | No |
| 51 | SMC1A |  |  | 885 | -0.367 | -0.5485 | No |
| 52 | TCF19 |  |  | 890 | -0.369 | -0.5480 | No |
| 53 | CKAP2 |  |  | 900 | -0.375 | -0.5517 | No |
| 54 | TK1 |  |  | 902 | -0.375 | -0.5485 | No |
| 55 | PARP2 |  |  | 912 | -0.379 | -0.5522 | Yes |
| 56 | TUBB4B |  |  | 916 | -0.380 | -0.5506 | Yes |
| 57 | TUBG1 |  |  | 917 | -0.381 | -0.5465 | Yes |
| 58 | HIST1H2AM |  |  | 918 | -0.381 | -0.5424 | Yes |
| 59 | DSN1 |  |  | 926 | -0.385 | -0.5443 | Yes |
| 60 | USP1 |  |  | 930 | -0.386 | -0.5427 | Yes |
| 61 | HAT1 |  |  | 933 | -0.387 | -0.5402 | Yes |
| 62 | PSIP1 |  |  | 934 | -0.388 | -0.5360 | Yes |
| 63 | DERA |  |  | 937 | -0.389 | -0.5335 | Yes |
| 64 | CDKN2D |  |  | 942 | -0.390 | -0.5327 | Yes |
| 65 | DIAPH3 |  |  | 943 | -0.390 | -0.5285 | Yes |
| 66 | TMEM237 |  |  | 953 | -0.396 | -0.5320 | Yes |
| 67 | DCBLD2 |  |  | 957 | -0.397 | -0.5303 | Yes |
| 68 | SKA3 |  |  | 965 | -0.399 | -0.5320 | Yes |
| 69 | HIRIP3 |  |  | 970 | -0.402 | -0.5311 | Yes |
| 70 | RTKN2 |  |  | 973 | -0.403 | -0.5284 | Yes |
| 71 | CEP78 |  |  | 984 | -0.408 | -0.5327 | Yes |
| 72 | AKAP12 |  |  | 994 | -0.412 | -0.5360 | Yes |
| 73 | SPDL1 |  |  | 995 | -0.412 | -0.5315 | Yes |
| 74 | CDC45 |  |  | 1000 | -0.413 | -0.5305 | Yes |
| 75 | CDH2 |  |  | 1007 | -0.418 | -0.5311 | Yes |
| 76 | CCDC34 |  |  | 1013 | -0.421 | -0.5309 | Yes |
| 77 | CMC2 |  |  | 1017 | -0.425 | -0.5289 | Yes |
| 78 | MIS18A |  |  | 1020 | -0.427 | -0.5260 | Yes |
| 79 | SAC3D1 |  |  | 1025 | -0.429 | -0.5248 | Yes |
| 80 | TPX2 |  |  | 1034 | -0.437 | -0.5269 | Yes |
| 81 | KIF22 |  |  | 1051 | -0.446 | -0.5359 | Yes |
| 82 | SMC2 |  |  | 1062 | -0.452 | -0.5397 | Yes |
| 83 | DEPDC1 |  |  | 1074 | -0.457 | -0.5442 | Yes |
| 84 | PSMC3IP |  |  | 1075 | -0.457 | -0.5393 | Yes |
| 85 | HES4 |  |  | 1079 | -0.460 | -0.5369 | Yes |
| 86 | MND1 |  |  | 1083 | -0.461 | -0.5345 | Yes |
| 87 | ATAD5 |  |  | 1091 | -0.465 | -0.5355 | Yes |
| 88 | ZWINT |  |  | 1099 | -0.467 | -0.5365 | Yes |
| 89 | UPP1 |  |  | 1101 | -0.469 | -0.5322 | Yes |
| 90 | SAT1 |  |  | 1109 | -0.473 | -0.5332 | Yes |
| 91 | SPARC |  |  | 1127 | -0.493 | -0.5425 | Yes |
| 92 | RBBP8 |  |  | 1128 | -0.494 | -0.5372 | Yes |
| 93 | ASF1B |  |  | 1133 | -0.498 | -0.5352 | Yes |
| 94 | PCNA |  |  | 1142 | -0.504 | -0.5367 | Yes |
| 95 | VRK1 |  |  | 1143 | -0.505 | -0.5312 | Yes |
| 96 | TTYH1 |  |  | 1144 | -0.505 | -0.5257 | Yes |
| 97 | CKAP2L |  |  | 1147 | -0.508 | -0.5219 | Yes |
| 98 | TMEM106C |  |  | 1148 | -0.509 | -0.5164 | Yes |
| 99 | HSPB11 |  |  | 1149 | -0.511 | -0.5109 | Yes |
| 100 | MXD3 |  |  | 1153 | -0.514 | -0.5079 | Yes |
| 101 | NUSAP1 |  |  | 1154 | -0.515 | -0.5023 | Yes |
| 102 | GMNN |  |  | 1155 | -0.516 | -0.4967 | Yes |
| 103 | DBF4 |  |  | 1157 | -0.520 | -0.4919 | Yes |
| 104 | MIS18BP1 |  |  | 1162 | -0.524 | -0.4897 | Yes |
| 105 | RRM1 |  |  | 1166 | -0.534 | -0.4865 | Yes |
| 106 | ORC6 |  |  | 1179 | -0.551 | -0.4909 | Yes |
| 107 | CENPU |  |  | 1182 | -0.555 | -0.4866 | Yes |
| 108 | CDCA3 |  |  | 1187 | -0.563 | -0.4839 | Yes |
| 109 | ASPM |  |  | 1189 | -0.567 | -0.4786 | Yes |
| 110 | FABP7 |  |  | 1190 | -0.568 | -0.4725 | Yes |
| 111 | FEN1 |  |  | 1191 | -0.569 | -0.4663 | Yes |
| 112 | NUF2 |  |  | 1194 | -0.574 | -0.4618 | Yes |
| 113 | DHFR |  |  | 1197 | -0.576 | -0.4573 | Yes |
| 114 | CKS1B |  |  | 1198 | -0.577 | -0.4510 | Yes |
| 115 | CLU |  |  | 1207 | -0.592 | -0.4515 | Yes |
| 116 | GTSE1 |  |  | 1210 | -0.602 | -0.4467 | Yes |
| 117 | CLSPN |  |  | 1212 | -0.605 | -0.4410 | Yes |
| 118 | BEX1 |  |  | 1216 | -0.615 | -0.4369 | Yes |
| 119 | CENPK |  |  | 1220 | -0.619 | -0.4328 | Yes |
| 120 | DUT |  |  | 1223 | -0.633 | -0.4277 | Yes |
| 121 | SMC4 |  |  | 1224 | -0.638 | -0.4207 | Yes |
| 122 | MYBL2 |  |  | 1225 | -0.639 | -0.4138 | Yes |
| 123 | CKS2 |  |  | 1226 | -0.641 | -0.4068 | Yes |
| 124 | ANLN |  |  | 1229 | -0.646 | -0.4016 | Yes |
| 125 | CTGF |  |  | 1234 | -0.654 | -0.3979 | Yes |
| 126 | CENPA |  |  | 1243 | -0.679 | -0.3975 | Yes |
| 127 | LYPD1 |  |  | 1248 | -0.698 | -0.3933 | Yes |
| 128 | AURKB |  |  | 1250 | -0.700 | -0.3866 | Yes |
| 129 | MELK |  |  | 1251 | -0.701 | -0.3790 | Yes |
| 130 | CCNA2 |  |  | 1255 | -0.716 | -0.3738 | Yes |
| 131 | UBE2T |  |  | 1256 | -0.719 | -0.3660 | Yes |
| 132 | CDCA2 |  |  | 1258 | -0.727 | -0.3590 | Yes |
| 133 | ARHGAP11A |  |  | 1262 | -0.735 | -0.3536 | Yes |
| 134 | HMGB2 |  |  | 1263 | -0.736 | -0.3456 | Yes |
| 135 | RAD51AP1 |  |  | 1264 | -0.741 | -0.3376 | Yes |
| 136 | KIF23 |  |  | 1265 | -0.741 | -0.3295 | Yes |
| 137 | KIFC1 |  |  | 1267 | -0.748 | -0.3223 | Yes |
| 138 | TACC3 |  |  | 1268 | -0.751 | -0.3141 | Yes |
| 139 | TYMS |  |  | 1272 | -0.770 | -0.3084 | Yes |
| 140 | ATAD2 |  |  | 1275 | -0.775 | -0.3017 | Yes |
| 141 | ID3 |  |  | 1276 | -0.785 | -0.2932 | Yes |
| 142 | NDC80 |  |  | 1278 | -0.810 | -0.2852 | Yes |
| 143 | IFITM3 |  |  | 1279 | -0.812 | -0.2764 | Yes |
| 144 | SYNE2 |  |  | 1282 | -0.829 | -0.2692 | Yes |
| 145 | ESCO2 |  |  | 1283 | -0.846 | -0.2600 | Yes |
| 146 | CALM2 |  |  | 1286 | -0.878 | -0.2522 | Yes |
| 147 | HIST2H2AC |  |  | 1288 | -0.880 | -0.2435 | Yes |
| 148 | MALAT1 |  |  | 1289 | -0.884 | -0.2339 | Yes |
| 149 | HJURP |  |  | 1290 | -0.897 | -0.2242 | Yes |
| 150 | SPC25 |  |  | 1294 | -0.932 | -0.2166 | Yes |
| 151 | RRM2 |  |  | 1295 | -0.933 | -0.2065 | Yes |
| 152 | KPNA2 |  |  | 1297 | -0.972 | -0.1968 | Yes |
| 153 | FBXO5 |  |  | 1301 | -0.992 | -0.1887 | Yes |
| 154 | NCAPG |  |  | 1303 | -1.015 | -0.1785 | Yes |
| 155 | HIST1H2AH |  |  | 1305 | -1.040 | -0.1681 | Yes |
| 156 | CDKN1A |  |  | 1306 | -1.046 | -0.1567 | Yes |
| 157 | IGFBP5 |  |  | 1307 | -1.052 | -0.1453 | Yes |
| 158 | HIST1H1E |  |  | 1309 | -1.319 | -0.1319 | Yes |
| 159 | HIST1H1C |  |  | 1310 | -1.391 | -0.1168 | Yes |
| 160 | CDK1 |  |  | 1311 | -1.433 | -0.1012 | Yes |
| 161 | UBE2C |  |  | 1312 | -1.466 | -0.0853 | Yes |
| 162 | TOP2A |  |  | 1313 | -1.528 | -0.0687 | Yes |
| 163 | HIST1H1B |  |  | 1318 | -1.922 | -0.0513 | Yes |
| 164 | HIST1H1D |  |  | 1319 | -1.958 | -0.0301 | Yes |
| 165 | HIST1H4C |  |  | 1320 | -2.769 | 0.0000 | Yes |
Table: GSEA details [plain text format]

  

Fig 2: FELDMAN\_2020\_S-G2: Random ES distribution      
 Gene set null distribution of ES for **FELDMAN\_2020\_S-G2**
